# Existence and Uniqueness Theorems for Impulsive Fractional Differential Equations with the Two-Point and Integral Boundary Conditions

**DOI:** 10.1155/2014/918730

**Published:** 2014-03-23

**Authors:** M. J. Mardanov, N. I. Mahmudov, Y. A. Sharifov

**Affiliations:** ^1^Institute of Mathematics and Mechanics, ANAS, B. Vahabzade Street 9, 1141 Baku, Azerbaijan; ^2^Department of Mathematics, Eastern Mediterranean University, Gazimagusa, North Cyprus, Mersin 10, Turkey; ^3^Institute of Cybernetics, ANAS, B. Vahabzade Street 9, 1141 Baku, Azerbaijan; ^4^Baku State University, Z. Khalilov Street 23, 1148 Baku, Azerbaijan

## Abstract

We study a boundary value problem for the system of nonlinear impulsive fractional differential equations of order **α** (0 < **α** ≤ 1) involving the two-point and integral boundary conditions. Some new results on existence and uniqueness of a solution are established by using fixed point theorems. Some illustrative examples are also presented. We extend previous results even in the integer case **α** = 1.

## 1. Introduction

For the last decades, fractional calculus has received a great attention because fractional derivatives provide an excellent tool for the description of memory and hereditary properties of various processes of science and engineering. Indeed, we can find numerous applications in viscoelasticity [1–3], dynamical processes in self-similar structures [4], biosciences [5], signal processing [6], system control theory [7], electrochemistry [8], and diffusion processes [9].

On the other hand, the study of dynamical systems with impulsive effects has been an object of intensive investigations in physics, biology, engineering, and so forth. The interest in the study of them is that the impulsive differential systems can be used to model processes which are subject to abrupt changes and which cannot be described by the classical differential problems (e.g., see [10–13] and references therein). Cauchy problems, boundary value problems, and nonlocal problems for impulsive fractional differential equations have been attractive to many researchers; one can see [10–22] and references therein.

Fečkan et al. [22] investigated the existence and uniqueness of solutions for

(1) D0+αcx(t)=f(t,x(t)), t∈J′≔J∖{t1,…,tp},J:=[0,T],x(ti+)−x(ti−)=ai, i=1,2,…,p,x(0)=x0, ak∈R,

where ^*c*^ *D*_0+_^*α*^ denotes the Caputo fractional derivative of order *α* ∈ (0,1) and *f* : *J* × **R** → **R** is a given continuous function.

In [21], Guo and Jiang discussed the existence of solutions for the following nonlinear fractional differential equations with boundary value conditions:

(2) D0+αcx(t)=f(t,x(t)), t∈J′≔J∖{t1,…,tp},J:=[0,T],x(ti+)−x(ti−)=Ii(x(ti−)), i=1,2,…,p,ax(0)+bx(T)=c,

where ^*c*^ *D*_0+_^*α*^ is the Caputo fractional derivative of order *α* ∈ (0,1) with the lower limit zero, *f* : *J* × **R** → **R** is jointly continuous, *t*_*k*_ satisfy 0 = *t* < *t*_1_ < ⋯<*t*_*p*_ < *t*_*p*+1_ = *T*, *x*(*t*_*k*_^+^) = lim⁡_*ε*→0^+^_ *x*(*t*_*k*_ + *ε*) and *x*(*t*_*k*_^−^) = lim⁡_*ε*→0^+^_ *x*(*t*_*k*_ − *ε*) represent the right and left limits of *x*(*t*) at *t* = *t*_*k*_, *I*_*k*_ ∈ *C*(**R**, **R**), and *a*, *b*, *c* are real constants with *a* + *b* ≠ 0.

Ashyralyev and Sharifov [20] considered nonfractional *n*-dimensional analogues of the problem (2) with two-point and integral boundary conditions.

Motivated by the papers above, in this paper, we study impulsive fractional differential equations with the two-point and integral boundary conditions in the following form:

(3) cD0+αx(t)=f(t,x(t)), t∈J′,x(tj+)−x(tj)=Ij(x(tj)), j=1,2,…,p,Ax(0)+Bx(T)=∫0Tg(s,x(s))ds,

where *A*, *B* ∈ **R** ^*n*×*n*^ are given matrices and det(*A* + *B*)≠0. Here *f*, *g* : [0, *T*] × **R** ^*n*^ → **R** ^*n*^ and *I*_*i*_ : **R** ^*n*^ → **R** ^*n*^ are given functions.

The rest of the paper is organized as follows. In Section 2, we give some notations, recall some concepts, and introduce a concept of a piecewise continuous solution for our problem. In Section 3, we give two main results: the first result based on the Banach contraction principle and the second result based on the Schaefer fixed point theorem. Some examples are given in Section 4 to demonstrate the application of our main results.

## 2. Preliminaries

In this section, we introduce notations, definitions, and preliminary facts that will be used in the remainder of this paper. By *C*(*J*, **R** ^*n*^) we denote the Banach space of all continuous functions from *J* to **R** ^*n*^ with the norm

(4) $$\begin{array}{c} {||x||}_{C}=\max\left\{\left|x\left(t\right)\right|:t\in J\right\}, \end{array}$$

where |·| is the norm in space **R** ^*n*^. We also introduce the Banach space

(5) PC(J,Rn)={x:J⟶Rn:x(t)∈C((ti,ti+1],Rn),   i=0,1,2…,p,  x(ti−)and  x(ti+)   exist  i=1,…,p,  and  x(ti−)=x(ti)},

with the norm

(6) $$\begin{array}{c} {||x||}_{PC}:=\sup\left\{\left|x\left(t\right)\right|:t\in J\right\}. \end{array}$$

If *A* ∈ **R** ^*n*×*n*^, then ||*A*|| is the norm of *A*.

Let us recall the following known definitions and results. For more details see [15, 16].

**Definition 1**

If *g* ∈ *C*[*a*, *b*] and *α* > 0, then the Riemann-Liouville fractional integral is defined by

(7) $$\begin{array}{c} I_{a+}^{\alpha }g\left(t\right)=\frac{1}{\Gamma \left(\alpha \right)}\overset{t}{\underset{a}{\int }}\frac{g\left(s\right)}{{\left(t-s\right)}^{1-\alpha }}ds, \end{array}$$

where Γ(·) is the Gamma function defined for any complex number *z* as

(8) $$\begin{array}{c} \Gamma \left(z\right)=\overset{\infty }{\underset{0}{\int }}t^{z-1}e^{-t}dt. \end{array}$$

**Definition 2**

The Caputo fractional derivative of order *α* > 0 of a continuous function *g* : [*a*, *b*] → **R** is defined by

(9) $$\begin{array}{c} {}^{c}D_{a+}^{\alpha }g\left(t\right)=\frac{1}{\Gamma \left(n-\alpha \right)}\overset{t}{\underset{a}{\int }}\frac{g^{\left(n\right)}\left(s\right)}{{\left(t-s\right)}^{\alpha -n+1}}ds, \end{array}$$

where *n* = [*α*] + 1 (the notation [*α*] stands for the largest integer not greater than *α*).

**Remark 3**

Under natural conditions on *g*(*t*), the Caputo fractional derivative becomes the conventional integer order derivative of the function *g*(*t*) as *α* → *n*.

**Remark 4**

Let *α*, *β* > 0 and *n* = [*α*] + 1; then the following relations hold:

(10) D0+αctβ=Γ(β)Γ(β−α)tβ−1, β>n,D0+αctk=0, k=0,1,2,…,n−1.

**Lemma 5**

For *α* > 0, *g*(*t*) ∈ *C*[0, *T*]⋂*L*_1_[0, *T*], the homogeneous fractional differential equation,

(11) $$\begin{array}{c} {}^{c}D_{0+}^{\alpha }g\left(t\right)=0, \end{array}$$

has a solution

(12) $$\begin{array}{c} g\left(t\right)=c_{0}+c_{1}t+c_{2}t^{2}+\cdot \cdot \cdot +c_{n-1}t^{n-1}, \end{array}$$

where *c*_*i*_ ∈ **R**, *i* = 0,1,…, *n* − 1, and *n* = [*α*] + 1.

**Lemma 6**

Assume that *g*(*t*) ∈ *C*[0, *T*]⋂*L*_1_[0, *T*], with derivative of order *n* that belongs to *C*[0, *T*]⋂*L*_1_[0, *T*]; then

(13) I0+αD0+αcg(t)=g(t)+c0+c1t+c2t2+···+cn−1tn−1,

where *c*_*i*_ ∈ **R**, *i* = 0,1,…, *n* − 1, and *n* = [*α*] + 1.

**Lemma 7**

Let *p*, *q* ≥ 0, *f* ∈ *L*_1_[0, *T*]. Then

(14) $$\begin{array}{c} I_{0+}^{p}I_{0+}^{q}f\left(t\right)=I_{0+}^{p+q}f\left(t\right)=I_{0+}^{q}I_{0+}^{p}f\left(t\right) \end{array}$$

is satisfied almost everywhere on [0, *T*]. Moreover, if *f* ∈ *C*[0, *T*], then (14) is true for all *t* ∈ [0, *T*].

**Lemma 8**

If *α* > 0, *f* ∈ *C*([0, *T*]), then ^*c*^ *D*_0+_^*α*^ *I*_0+_^*α*^ *f*(*t*) = *f*(*t*) for all *t* ∈ [0, *T*].

We define a solution problem (3) as follows.

**Definition 9**

A function *x* ∈ *PC*(*J*, **R** ^*n*^) is said to be a solution of problem (3) if ^*c*^ *D*_0+_^*α*^ *x*(*t*) = *f*(*t*, *x*(*t*)), for *t* ∈ [0, *T*], *t* ≠ *t*_*i*_, *i* = 1,2,…, *p*, and for each *i* = 1,2,…, *p*, *x*(*t*_*i*_^+^) − *x*(*t*_*i*_) = *I*_*i*_(*x*(*t*_*i*_)), 0 = *t*_0_ < *t*_1_ < *t*_2_ < ⋯<*t*_*p*_ < *t*_*p*+1_ = *T*, and the boundary conditions *Ax*(0) + *Bx*(*T*) = ∫_0_^*T*^ *g*(*s*, *x*(*s*))*ds* are satisfied.

We have the following result which is useful in what follows.

**Theorem 10**

Let *f*, *g* ∈ *C*(*J*, **R** ^*n*^). Then the function *x* is a solution of the boundary value problem for impulsive differential equation

(15) cD0+αx(t)=f(t), t∈J′,x(tj+)−x(tj)=Ij(x(tj)), j=1,2,…,p,Ax(0)+Bx(T)=∫0Tg(s)ds

if and only if

(16) x(t)=(A+B)−1∫0Tg(s)ds +∑0<tj<TK(tk,tj)Ij(x(tj−)) +1Γ(α)∑0<tj≤TK(tk,tj)∫tj−1tj(tj−s)α−1f(s)ds +1Γ(α)∫tkt(t−s)α−1f(s)ds, tk<t≤tk+1,

where

(17) K(t,τ)={0,t=0,(A+B)−1A,0<τ≤t,−(A+B)−1B,t<τ≤T.

**Proof**

Assume that *x* is a solution of the boundary value problem (15); then we have

(18) $$\begin{array}{c} x\left(t\right)=x\left(0\right)+\frac{1}{\Gamma \left(\alpha \right)}\overset{t}{\underset{0}{\int }}{\left(t-s\right)}^{\alpha -1}f\left(s\right)ds,\;0\leq t\leq t_{1}. \end{array}$$

If *t*_1_ < *t* ≤ *t*_2_, then

(19) cD0+αx(t)=f(t), t1<t≤t2,x(t1+)−x(t1)=I1(x(t1)).

Integrating the expression (19) from *t*_1_ to *t*, one can obtain

(20) $$\begin{array}{c} x\left(t\right)=x\left(t_{1}^{+}\right)+\frac{1}{\Gamma \left(\alpha \right)}\overset{t}{\underset{t_{1}}{\int }}{\left(t-s\right)}^{\alpha -1}f\left(s\right)ds. \end{array}$$

It follows that

(21) x(t)=x(t1)+I1(x(t1))+1Γ(α)∫t1t(t−s)α−1f(s)ds=x(0)+I1(x(t1))+1Γ(α)∫0t1(t1−s)α−1f(s)ds +1Γ(α)∫t1t(t−s)α−1f(s)ds.

Thus if *t* ∈ (*t*_*k*_, *t*_*k*+1_], we get

(22) x(t)=x(tk)+Ik(x(tk))+1Γ(α)∫tkt(t−s)α−1f(s)ds=x(0)+∑0<tk<tIk(x(tk)) +1Γ(α)∑0<tk<t∫tk−1tk(tk−s)α−1f(s)ds +1Γ(α)∫tkt(t−s)α−1f(s)ds,

where *x*(0) is still an arbitrary constant vector. For determining *x*(0) we use the boundary value condition *Ax*(0) + *Bx*(*T*) = ∫_0_^*T*^ *g*(*s*)*ds*:

(23) ∫0Tg(s)ds=Ax(0)+Bx(T)=(A+B)x(0)+B∑0<tk<TIk(x(tk)) +1Γ(α)∑0<tk<TB∫tk−1tk(tk−s)α−1f(s)ds +1Γ(α)B∫tkT(T−s)α−1f(s)ds.

Hence, we obtain

(24) x(0)=(A+B)−1∫0Tg(s)ds−(A+B)−1B∑0<tj<TIj(x(tj)) −1Γ(α)(A+B)−1B∑0<tj≤T∫tj−1tj(tj−s)α−1f(s)ds,

and consequently for all *t* ∈ (*t*_*k*_, *t*_*k*+1_]

(25) x(t)=(A+B)−1∫0Tg(s)ds−(A+B)−1B∑0<tj<TIj(x(tj)) −1Γ(α)(A+B)−1B∑0<tj≤T∫tj−1tj(tj−s)α−1f(s)ds +∑0<tj<tIj(x(tj)) +1Γ(α)∑0<tj<t∫tj−1tj(tj−s)α−1f(s)ds +1Γ(α)∫tkt(t−s)α−1f(s)ds=(A+B)−1∫0Tg(s)ds−(A+B)−1B∑tk<tj<TIj(x(tj)) +(A+B)−1A∑0<tj<tIj(x(tj)) −1Γ(α)(A+B)−1B∑tk<tj≤T∫tj−1tj(tj−s)α−1f(s)ds +1Γ(α)(A+B)−1A∑0<tj<t∫tj−1tj(tj−s)α−1f(s)ds +1Γ(α)∫tkt(t−s)α−1f(s)ds=(A+B)−1∫0Tg(s)ds+∑0<tj<TK(tk,tj)Ij(x(tj−)) +1Γ(α)∑0<tj≤TK(tk,tj)∫tj−1tj(tj−s)α−1f(s)ds +1Γ(α)∫tkt(t−s)α−1f(s)ds.

Conversely, assume that *x* satisfies (16). If *t* ∈ [0, *t*_1_], then, using the fact that ^*c*^ *D*_0^+^_^*α*^ is the left inverse of *I*_0+_^*α*^, we get ^*c*^ *D*_0+_^*α*^ *x*(*t*) = *f*(*t*), *t*_0_ < *t* ≤ *t*_1_. If *t* ∈ (*t*_*k*_, *t*_*k*+1_], *k* = 1,2,…, *p*, then, using the fact that the Caputo derivative of a constant is equal to zero, we obtain ^*c*^ *D*_0+_^*α*^ *x*(*t*) = *f*(*t*), *t*_*k*_ < *t* ≤ *t*_*k*+1_, and *x*(*t*_*k*_^+^) − *x*(*t*_*k*_) = *I*_*k*_(*x*(*t*_*k*_)). The lemma is proved.

**Theorem 11 (see [18])**

Let *X* be a Banach space and *W* ⊂ *PC*(*J*, *X*). If the following conditions are satisfied,

1. *W* is uniformly bounded subset of *PC*(*J*, *X*),
2. *W* is equicontinuous in (*t*_*k*_, *t*_*k*+1_), *k* = 0,1, 2,…, *p*, where *t*_0_ = 0, *t*_*p*+1_ = *T*,
3. *W*(*t*) = {*u*(*t*) : *u* ∈ *W*, *t* ∈ *J*′}, *W*(*t*_*k*_^+^) = {*u*(*t*_*k*_^+^) : *u* ∈ *W*}, and *W*(*t*_*k*_^−^) = {*u*(*t*_*k*_^−^) : *u* ∈ *W*} are relatively compact subsets of *X*,

then *W* is a relatively compact subset of *PC*(*J*, *X*).

## 3. Main Results

Our first result is based on Banach fixed point theorem. Before stating and proving the main results, we introduce the following hypotheses.

(H1) *f*, *g* : *J* × **R** ^*n*^ → **R** ^*n*^ are continuous functions.
(H2) There are constants *L*_*f*_ > 0 and *L*_*g*_ > 0 such that
  (26) $$\begin{array}{c} \left|f\left(t,x\right)-f\left(t,y\right)\right|\leq L_{f}\left|x-y\right|,\;\\ \left|g\left(t,x\right)-g\left(t,y\right)\right|\leq L_{g}\left|x-y\right| \end{array}$$
 for each *t* ∈ [0, *T*] and all *x*, *y* ∈ **R** ^*n*^.
(H3) There exist constants *l*_*i*_ > 0, *i* = 1,2,…, *p* such that
  (27) $$\begin{array}{c} \left|I_{i}\left(x\right)-I_{i}\left(y\right)\right|\leq l_{i}\left|x-y\right| \end{array}$$
 for all *x*, *y* ∈ **R** ^*n*^.

For brevity, let

(28) $$\begin{array}{c} L_{AB}:=\max\left(||{\left(A+B\right)}^{-1}A||,\;||{\left(A+B\right)}^{-1}B||\right). \end{array}$$

**Theorem 12**

Assume that (H1)–(H3) hold. If

(29) Lg||(A+B)−1||T+LAB∑j=1plj  +1Γ(α+1)LfLAB∑j=1p+1(tj−tj−1)α  +TαΓ(α+1)Lf<1,

then the boundary value problem (3) has a unique solution on *J*.

**Proof**

The proof is based on the classical Banach fixed theorem for contractions. Let us set

(30) sup⁡t∈J⁡|f(t,0)|=Mf,  sup⁡t∈J|g(t,0)|=Mg,|Ik(0)|=mk,δ(T):=Lg||(A+B)−1||T+LAB∑j=1plj +1Γ(α+1)LfLAB∑j=1p+1(tj−tj−1)α +TαΓ(α+1)Lf<1,γ:=||(A+B)−1||TMg+LAB∑j=1pmj +1Γ(α+1)MfLAB∑j=1p+1(tj−tj−1)α+TαΓ(α+1)Mf.

It is clear that

(31) |f(t,x)|≤Mf+Lf|x|,|g(t,x)|≤Mg+Lg|x|,|Ik(x)|≤mk+lk|x|, t∈J,  x∈Rn,||K(tk,tj)||≤LAB, k,j=0,1,…,p+1.

Consider

(32) $$\begin{array}{c} B_{r}:=\left\{x\in PC\left(J,R^{n}\right):{||x||}_{PC}\leq r\right\}, \end{array}$$

where

(33) $$\begin{array}{c} r\geq \frac{\gamma }{1-\delta \left(T\right)}. \end{array}$$

Let *Q* be the following operator:

(34) (Qx)(t)={(A+B)−1∫0Tg(s)ds  +∑0<tj<TK(tk,tj)Ij(x(tj)) +1Γ(α)∑0<tj≤TK(tk,tj)∫tj−1tj(tj−s)α−1f(s)ds +1Γ(α)∫tkt(t−s)α−1f(s)ds, tk<t≤tk+1,                   k=1,…,p.

We show that *Q* maps *B*_*r*_ into *B*_*r*_. It is clear that *Q* is well defined on *PC*(*J*, **R** ^*n*^). Moreover for *x* ∈ *B*_*r*_ and *t* ∈ (*t*_*k*_, *t*_*k*+1_], *k* = 0,…, *p*, we have

(35) |(Qx)(t)|≤||(A+B)−1||∫0T|g(s,x(s))|ds +∑0<tj<T||K(tk,tj)|||Ij(x(tj))| +1Γ(α)∑0<tj≤T||K(tk,tj)|| ×∫tj−1tj(tj−s)α−1|f(s,x(s))|ds +1Γ(α)∫tkt(t−s)α−1|f(s,x(s))|ds≤||(A+B)−1||T(Mg+Lgr)+LAB∑j=1p(mj+ljr) +1αΓ(α)LAB(Mf+Lfr)∑j=1p+1(tj−tj−1)α +TααΓ(α)(Mf+Lfr)=γ+δ(T)r≤r.

Consequently *Q* maps *PC*(*J*, **R** ^*n*^) into itself.

Next we will show that *Q* is a contraction. Let *x*, *y* ∈ *PC*(*J*, **R** ^*n*^). Then, for each *t* ∈ (*t*_*k*_, *t*_*k*+1_], *k* = 0,…, *p*, we have

(36) |(Qx)(t)−(Qy)(t)| ≤||(A+B)−1||∫0T|g(s,x(s))−g(s,y(s))|ds  +∑0<tj<T||K(tk,tj)|||Ij(x(tj))−Ij(y(tj))|  +1Γ(α)∑0<tj≤T||K(tk,tj)||  ×∫tj−1tj(tj−s)α−1|f(s,x(s))−f(s,y(s))|ds  +1Γ(α)∫tkt(t−s)α−1|f(s,x(s))−f(s,y(s))|ds ≤Lg||(A+B)−1||∫0T|x(s)−y(s)|ds  +LAB∑j=1p|Ij(x(tj))−Ij(y(tj))|  +LfLABΓ(α)∑0<tj≤T∫tj−1tj(tj−s)α−1|x(s)−y(s)|ds  +LfΓ(α)∫tkt(t−s)α−1|x(s)−y(s)|ds ≤(Lg||(A+B)−1||T+LAB∑j=1plj    +1αΓ(α)LfLAB∑j=1p+1(tj−tj−1)α+TααΓ(α)Lf)  ×||x−y||PC.

Thus, *Q* is a contraction mapping on *PC*(*J*, **R** ^*n*^) due to condition (29) and the operator *Q* has a unique fixed point on *PC*(*J*, **R** ^*n*^) which is a unique solution to (3).

The second result is based on the Schaefer fixed point theorem. We introduce the following assumptions.

(H4) There exist constants *N*_*f*_ > 0, *N*_*g*_ > 0 such that |*f*(*t*, *x*)| ≤ *N*_*f*_, |*g*(*t*, *x*)| ≤ *N*_*g*_ for each *t* ∈ *J* and all *x* ∈ **R** ^*n*^.
(H5) *I*_*k*_ ∈ *C*(**R** ^*n*^, **R** ^*n*^).

**Theorem 13**

Assume that (H1), (H4), and (H5) hold. Then the boundary value problem (3) has at least one solution on *J*.

**Proof**

We will use Schaefer's fixed point theorem to prove that *Q* defined by (34) has a fixed point. The proof will be given in several steps.

*Step  1*. Operator *Q* is continuous.

Let {*x*_*n*_} be a sequence such that *x*_*n*_ → *x* in *PC*(*J*, **R** ^*n*^). Then, for each *k* = 0,1, 2,…, *p* and for all *t* ∈ (*t*_*k*_, *t*_*k*+1_], we have

(37) |(Qx)(t)−(Qxn)(t)| ≤||(A+B)−1||∫0T|g(s,x(s))−g(s,xn(s))|ds  +∑0<tj<T||K(tk,tj)|||Ij(x(tj))−Ij(xn(tj))|  +1Γ(α)∑0<tj≤T||K(tk,tj)||  ×∫tj−1tj(tj−s)α−1|f(s,x(s))−f(s,xn(s))|ds  +1Γ(α)∫0t(t−s)α−1|f(s,x(s))−f(s,xn(s))|ds.

Since *f*, *g*, and *I*_*k*_, *k* = 0,1, 2,…, *p*, are continuous functions, we have

(38) $$\begin{array}{c} {||Qx_{n}-Qx||}_{PC}⟶0 \end{array}$$

as *n* → *∞*.

*Step  2*. *Q* maps bounded sets in bounded sets in *PC*(*J*, **R** ^*n*^).

Indeed, it is enough to show that, for any *η* > 0, there exists a positive constant *l* such that, for each *x* ∈ *B*_*η*_ = {*x* ∈ *PC*(*J*, **R** ^*n*^) : ||*x*||_*PC*_ ≤ *η*}, we have ||*Q*(*x*)||_*PC*_ ≤ *l*. By (H4), (H5) we have, for each *k* = 1,2,…, *p* and for all *t* ∈ (*t*_*k*_, *t*_*k*+1_],

(39) |(Qx)(t)| ≤||(A+B)−1||∫0T|g(s,x(s))|ds  +∑0<tj<T||K(tk,tj)|||Ij(x(tj))|  +1Γ(α)∑0<tj≤T||K(tk,tj)||∫tj−1tj(tj−s)α−1|f(s,x(s))|ds  +1Γ(α)∫tkt(t−s)α−1|f(s,x(s))|ds ≤||(A+B)−1||TNg+LAB∑j=1p|Ij(x(tj))|  +1αΓ(α)LABNf∑j=1p+1(tj−tj−1)α+TααΓ(α)Nf:=l.

Thus

(40) $$\begin{array}{c} {||Qx||}_{PC}\leq l. \end{array}$$

*Step  3*. *Q* maps bounded sets into equicontinuous sets of *PC*(*J*, **R** ^*n*^).

Let *τ*_1_, *τ*_2_ ∈ (*t*_*k*_, *t*_*k*+1_], *τ*_1_ < *τ*_2_, *B*_*η*_ be a bounded set of *PC*(*J*, **R** ^*n*^) as in Step  2, and let *x* ∈ *B*_*η*_. Then

(41) |(Qx)(τ2)−(Qx)(τ1)| ≤|1Γ(α)∫tiτ1[(τ2−s)α−1−(τ1−s)α−1]f(s,x(s))ds   +1Γ(α)∫τ1τ2(τ2−s)α−1f(s,x(s))ds| ≤NfΓ(α)∫tiτ1[(τ2−s)α−1−(τ1−s)α−1]ds  +NfΓ(α)∫τ1τ2(τ2−s)α−1ds ≤NfΓ(α+1)[2(τ2−τ1)α+τ2α−τ1α].

As *τ*_1_ → *τ*_2_, the right-hand side of the above inequality tends to zero.

As a consequence of Steps  1 to 3 together with the Arzela-Ascoli theorem (Theorem 11 with *X* = **R** ^*n*^), we can conclude that the operator *Q* : *PC*(*J*, **R** ^*n*^) → *PC*(*J*, **R** ^*n*^) is completely continuous.

*Step  4*. One has a priori bounds.

Now it remains to show that the set

(42) $$\begin{array}{c} \Delta =\left\{x\in PC\left(J,R^{n}\right):x=\lambda Q\left(x\right),\;\;\text{for}\;\text{some}\;0<\lambda <1\right\} \end{array}$$

is bounded.

Let then *x* = *λQ*(*x*) for some 0 < *λ* < 1. Thus, for each *t* ∈ (*t*_*k*_, *t*_*k*+1_], we have

(43) |x(t)|=|λ(Qx)(t)| ≤||(A+B)−1||∫0T|g(s,x(s))|ds  +∑0<tj<T||K(tk,tj)|||Ij(x(tj))|  +1Γ(α)∑0<tj≤T||K(tk,tj)||∫tj−1tj(tj−s)α−1|f(s,x(s))|ds  +1Γ(α)∫tkt(t−s)α−1|f(s,x(s))|ds ≤||(A+B)−1||TNg+LAB∑j=1p|Ij(x(tj))|  +1αΓ(α)LABNf∑j=1p+1(tj−tj−1)α+TααΓ(α)Nf.

Thus

(44) ||x||PC≤||(A+B)−1||TNg+LAB∑j=1p|Ij(x(tj))| +1αΓ(α)LABNf∑j=1p+1(tj−tj−1)α+TααΓ(α)Nf.

This shows that the set Δ is bounded. As a consequence of Schaefer's fixed point theorem, we deduce that *Q* has a fixed point which is a solution of the problem (3).

## 4. Examples

In this section, we give some examples to illustrate our main results.

**Example 1**

Consider

(45) D0+αcx1(t) =cos⁡(110  x2(t)), t∈(0,2)∖{1},D0+αcx2(t) =e−t9+et|x1(t)|1+|x1(t)|, t∈(0,2)∖{1},x1(0)+12x2(1)=0, x2(0)=1,Δx1(1)=110x2(1),    Δx2(1)=110x1(1)+5.

Consider boundary value problem (3) with *f*_1_(*t*, *x*_1_, *x*_2_) = cos⁡((1/10)*x*_2_(*t*)),  *f*_2_(*t*, *x*_1_, *x*_2_) = (*e* ^−*t*^/(9 + *e* ^*t*^))·(|*x*_1_|/(1 + |*x*_1_|)), and  *T* = 1,  *p* = 1,  $I_{1}\left(x_{1},x_{2}\right)=\left[\begin{array}{c} (1/10)x_{2}\\ (1/10)x_{1}+5 \end{array}\right]$.

Evidently,

(46) A=I=(1001),  B=(00.500),||(A+B)−1A||=||(1−1201)||=32,  ||(A+B)−1B||=||(01200)||=12,LAB=max⁡(32,12)=32,

and conditions (H1)–(H3) hold. We will show that condition (29) is satisfied for, say, *α* = 0,2. Indeed,

(47) LABl1+2α+1αΓ(α)LfLAB+2ααΓ(α)Lf =32×110+2αΓ(α+1)×310+2αΓ(α+1)×110 <32×110+54×25=1320<1,

where we used

(48) $$\begin{array}{c} \Gamma \left(\alpha +1\right)=\Gamma \left(1,2\right)=0.92,\;\;\frac{2^{\alpha }}{\Gamma \left(\alpha +1\right)}<\frac{1.15}{0.92}=1.25. \end{array}$$

Then, by Theorem 12, boundary value problem (45) has unique solution on [0,2].

**Example 2**

Consider

(49) D0+αcx1(t)=e−t9+et|x1(t)|1+x22(t), t∈(0,1),  t≠0,5D0+αcx2(t)=sinx1(t), t∈(0,1),  t≠0,5,x1(0)=1,  x2(0)+12x1(1)=0,Δx1(12)=11+x22(1/2),Δx2(12)=11+cos⁡2x1(1/2).

Here 0 < *α* ≤ 1,  *f*_1_(*t*, *x*_1_, *x*_2_) = *e* ^−*t*^/(1 + *x*_2_^2^(*t*)),  *f*_2_(*t*, *x*_1_, *x*_2_) = sin*x*_1_(*t*),  $A=\left(\begin{array}{cc} 1 & 0\\ 0 & 1 \end{array}\right)$,  $B=\left(\begin{array}{cc} 0 & 0\\ 0.5 & 0 \end{array}\right)$, and  *T* = 1,  *p* = 1,  $I_{1}(x_{1},x_{2})=\left[\begin{array}{c} 1/\left(1+x_{2}^{2}\right)\\ 1/\left(1+\cos^{2}x_{1}\right) \end{array}\right]$. Clearly, all the conditions of Theorem 13 are satisfied (*N*_*f*_ = 1, *N*_*g*_ = 0), and consequently boundary value problem (49) has at least one solution.
